# The Overlap of Poor Reading Comprehension in English and French

**DOI:** 10.3389/fpsyg.2020.00120

**Published:** 2020-02-05

**Authors:** Nadia D’Angelo, Klaudia Krenca, Xi Chen

**Affiliations:** ^1^Ontario Ministry of Education, Toronto, ON, Canada; ^2^Department of Applied Psychology & Human Development, Ontario Institute for Studies in Education, University of Toronto, Toronto, ON, Canada

**Keywords:** poor comprehenders, reading comprehension, French immersion, oral language skills, vocabulary, comprehension difficulties, bilingual learners

## Abstract

This study examined overlap and correlates of poor reading comprehension in English and French for children in early French immersion. Poor comprehenders were identified in grade 3 in English and French using a regression method to predict reading comprehension scores from age, non-verbal reasoning, word reading accuracy, and word reading fluency. Three groups of poor comprehenders were identified: 10 poor comprehenders in English and French, 11 poor comprehenders in English, and 10 poor comprehenders in French, and compared to 10 controls with good reading comprehension in both English and French. There was a moderate degree of overlap in comprehension difficulties in English and French among poor comprehenders with equivalent amounts of exposure to French, with a prevalence rate of 41.7% in our sample. Children who were poor comprehenders in both English and French consistently scored the lowest on English vocabulary in grade 1 and grade 3 and in French vocabulary in grade 3 suggesting that poor comprehenders’ vocabulary weaknesses in English as a primary language may contribute to comprehension difficulties in English and French.

## Introduction

There is considerable evidence to suggest that children who are at risk for reading difficulties in a second language (L2) can be identified through early assessment of word reading and cognitive skills in their first language (L1), before their oral language proficiency is fully developed in L2 (Geva and Clifton, 1994; Da Fontoura and Siegel, 1995; MacCoubrey et al., 2004). Much of this previous research is based on the premise that certain cognitive and linguistic skills, such as phonological processing, transfer across languages (e.g., Comeau et al., 1999; August and Shanahan, 2006). More recently, studies have investigated children’s reading comprehension difficulties that occur despite age-appropriate decoding skills (e.g., Nation et al., 2010; Tong et al., 2011). Relatively little is known about the identification of poor reading comprehension in the absence of poor decoding, and even less is known about whether reading comprehension difficulties manifest in a similar manner in L1 and L2 for children learning in a bilingual context. The present study aims to investigate overlap and early contributors of poor reading comprehension for children in early French immersion programs in Canada who receive school instruction in French, an additional language, while being exposed to English, their primary language of the community.

Reading comprehension is a complex process that involves the integration and coordination of various skills, including word decoding, the ability to decipher or recognize printed words, and oral language or listening comprehension, the ability to understand what is decoded in spoken form (Simple View of Reading; Gough and Tunmer, 1986). Most research into reading comprehension difficulties has focused on children with poor decoding whose weaknesses manifest early in reading development as phonological awareness and word reading deficits (e.g., Snowling, 2000). In contrast to poor decoders, poor comprehenders’ difficulties appear to emerge later, when decoding becomes automatized and more variance in reading comprehension is accounted for by oral language skills (Catts et al., 2012). Oral language difficulties tend to be masked by poor comprehenders’ age-appropriate decoding skills, and as a result, early indicators of later reading comprehension difficulties are often overlooked.

Existing longitudinal studies have used a retrospective approach to examine poor comprehenders’ deficits across previous grades and suggest that oral language weaknesses are prevalent in poor comprehenders before their reading comprehension difficulties become apparent (Catts et al., 2006; Nation et al., 2010; Tong et al., 2011). For example, Nation et al. (2010) identified poor comprehenders based on reading achievement at age 8 and retrospectively examined their reading and language skills beginning at age 5. While poor comprehenders’ phonological processing and word reading skills progressed over time, their oral language skills remained persistently weak, suggesting that early weaknesses in understanding and producing spoken language contributed to poor comprehenders’ comprehension difficulties.

The linguistic interdependence hypothesis suggests that L1 and L2 reading skills are interdependent, and that language and literacy skills acquired in one language facilitate reading development in the L2 (Cummins, 1984). Thus, it seems probable that the same cognitive and linguistic skills needed for successful reading comprehension in L1 contribute to reading development in L2 (e.g., Gottardo and Mueller, 2009; Mancilla-Martinez and Lesaux, 2010). Indeed, previous research suggests that it is possible to identify children at-risk for L2 reading difficulties based on their performance in L1 (Geva and Clifton, 1994; Da Fontoura and Siegel, 1995). However, few studies have investigated poor comprehenders in a bilingual context largely due to the complexity of understanding reading comprehension processes in L1 and L2. Children learning in an L2 are in the process of acquiring the language of instruction and it may be difficult to determine whether weaknesses in L2 reading comprehension reflect limited language learning experiences or are indicative of a language or reading impairment (Paradis et al., 2010; Li and Kirby, 2014; D’Angelo and Chen, 2017).

Li and Kirby (2014) examined the reading comprehension profiles of grade 8 emerging Chinese-English bilinguals in an English immersion program in China. Poor comprehenders were distinguished from average comprehenders based on their performance on English L2 vocabulary measures. The authors concluded that because the groups did not differ on Chinese L1 word reading and reading comprehension, poor comprehenders’ reading comprehension difficulties were due to limited English L2 proficiency. However, the comprehender groups in this study were selected using English L2 assessments only and therefore, children with an underlying oral language impairment across the two languages could not be identified. Since Chinese and English and are not closely related languages, vocabulary and reading comprehension may not have the same underlying mechanisms in each language.

A few studies have identified poor comprehenders based on English L1 reading performance in a French immersion context and suggest that poor comprehenders demonstrate relatively poor oral language skills in both English L1 and French L2 (e.g., D’Angelo et al., 2014; D’Angelo and Chen, 2017). D’Angelo et al. (2014) retrospectively investigated the reading and language abilities of a small sample of English L1 children in French immersion who were identified as poor and average comprehenders based on their English L1 reading performance in grade 3. They found that poor comprehenders scored relatively lower on English and French vocabulary across grades 1 to 3, despite average phonological awareness and word reading skills in both languages. Such findings suggest that poor comprehenders may indeed have an underlying problem in oral language. The current study extends the existing research to a larger, more representative sample of children in French immersion to facilitate comparison. The purpose is to determine the extent to which those identified as having poor reading comprehension in English, the societal language, also demonstrate poor reading comprehension in French, an additional language and the language of instruction.

Studies that have examined the co-occurrence of reading difficulties between an L1 and L2 have primarily focused on poor readers and suggest that there is some overlap of reading difficulty in L1 and L2 (Manis and Lindsey, 2010; McBride-Chang et al., 2013; Tong et al., 2015; Shum et al., 2016). For example, Manis and Lindsey (2010) found that 55% of grade 5 children who met the criteria for reading difficulties in English L2 (decoding scores at or below the 25^th^ percentile) were also identified with reading difficulties in Spanish L1. Similarly, McBride-Chang et al. (2013) tested the overlap of poor readers in Chinese L1 and English L2 (defined as those at or below the 25^th^ percentile on Chinese and English word reading tests) among 8-year-old children in Beijing and found that 40% of poor readers in Chinese L1 were also poor readers in English L2. In each study, children who were identified as poor readers in both languages scored lower on cognitive and linguistic tasks than children who were poor readers in only one language. On the other hand, children with poor reading in one language did not necessarily have difficulties in the other. It appears that the degree of overlap between poor reading is increased when the two languages are more closely related. However, these studies focused on the overlap status of poor readers based on poor decoding. We were interested in whether such overlap occurs for poor comprehenders who show discrepancies between their reading comprehension and decoding skills.

Only one known study at this time has explored the overlap between L1 and L2 reading comprehension difficulties. Tong et al. (2017) examined the co-occurrence of reading comprehension difficulties and associated longitudinal correlates in 10-year-old children with poor reading comprehension (defined as those at or below the 25^th^ percentile on reading comprehension tasks) in Chinese L1 and English L2. The authors found that approximately half (53%) of children with poor reading comprehension in Chinese L1 also experienced poor reading comprehension in English L2. Results indicated that word reading and language skills were longitudinal correlates of poor reading comprehension in Chinese and English. This study was among the first to investigate overlap of reading comprehension difficulties in L1 and L2 and to retrospectively examine sources of poor reading comprehension. However, the selection method used in this study identified poor comprehenders based on reading comprehension scores only and did not distinguish between children with poor oral language skills from those with poor decoding skills. In the present study, we aimed to understand the overlap of poor reading comprehension in English and French in the absence of decoding problems.

Given the challenges associated with defining poor reading comprehension in an additional language, the goal of the present study was to extend previous research on reading comprehension difficulties to English–French bilinguals to answer two specific research questions.

First, we asked whether children identified as poor comprehenders in English are also identified as poor comprehenders in French. Whereas most previous studies have examined overlap with word reading and reading comprehension scores at or below an arbitrary cut-off score, we utilized a regression technique to identify poor comprehenders in English and French by examining associations between reading comprehension scores, age, non-verbal reasoning, word reading accuracy, and word reading fluency. This approach defines groups more precisely than the cut-off score method because it examines relative discrepancies between various skills related to reading comprehension by distinguishing poor comprehenders from average and good comprehenders (e.g., Tong et al., 2011, 2014; Li and Kirby, 2014; D’Angelo and Chen, 2017).

Second, we asked what reading and language skills distinguish between poor comprehenders in English and French, poor comprehenders in English, and poor comprehenders in French. We anticipated that children identified as poor comprehenders in both English and French would show early and persistent oral language difficulties in both languages. English and French share many similarities in vocabulary, morphology, and syntax (e.g., LeBlanc and Seguin, 1996; Roy and Labelle, 2007; D’Angelo and Chen, 2017; D’Angelo et al., 2017). Both are represented by the Roman alphabet and an opaque writing system (Seymour et al., 2003). These shared structural properties are thought to facilitate cross-language associations between two languages (Koda, 2008). Therefore, we expected to see similar characteristics of reading comprehension difficulties between the two languages.

The socio-linguistic and educational context of the current study makes it possible to assess and compare English and French reading outcomes among children acquiring both languages. In Canada, French immersion is an additive dual language program that promotes oral and written language proficiency in both English and French, the official languages. Children in early French immersion programs are non-francophones who receive integrated language and content instruction primarily in French beginning in kindergarten or grade 1. However, these children often live in predominantly English-speaking environments with limited opportunity to hear and speak French outside of the classroom. Thus, French immersion classrooms are comprised of English-speaking children for whom French is the L2 and minority language children for whom English is the L2 and French the L3. English language arts instruction is generally introduced in grade 4.

Since the children in this study had similar and limited levels of French proficiency upon school entry, any differences in French reading and language abilities between children would be unlikely a result of differences in the amount of exposure the children had to French. Specifically, for children with poor reading comprehension in both English and French, we could be confident that weaknesses in oral language reflect a pervasive language impairment rather than a less developed French proficiency.

## Materials and Methods

### Participants

Participants were 180 children consisting of 83 males and 97 females who were recruited from early French immersion schools in a large Canadian city and tested in English and French in the spring of grade 1 (*M*_*age*_ = 80.36 months, *SD* = 4.18) and grade 3 (*M*_*age*_ = 104.66 months, *SD* = 4.06). As part of the inclusion criteria, children selected for this study were non-native speakers of French receiving school instruction entirely in French since school entry. Out of the 180 children, 135 (75%) spoke English as a primary language. Forty-five children (25%) were exposed to additional languages at home.

### Measures

The data in this study are from longitudinal research, in which several reading-related tasks were administered to participants between grades 1 and 3. Trained research assistants, who were fluent in the respective test language, administered tasks to participants at school. English and French instructions were used for French measures to ensure comprehension of the task. The order of the sessions was counterbalanced across participants and within each session the order of the task administration was randomized. Due to limited testing time, not all the same tasks were administered in each year of the study.

#### Non-verbal Reasoning

Children were administered the reasoning by analogy subtest of the Matrix Analogies Test in English to assess non-verbal reasoning in grade 1 (expanded form; Naglieri, 1985). For each item, children were asked to complete a figural matrix by choosing the missing piece from 5 to 6 possible choices. There were 16 items and testing was discontinued after four consecutive errors.

#### Phonological Awareness

This task was measured in grade 1 using the elision subtest of the Comprehensive Test of Phonological Processing (CTOPP; Wagner et al., 1999, 2013). The examiner read individual words aloud and children were asked to delete a syllable or phoneme from each word (e.g., “say *time* without saying/*m*/”). There were 34 test items presented in order of increasing difficulty. Testing was discontinued after three consecutive errors.

A parallel measure was created to assess phonological awareness in French. Twenty-six items were selected to match characteristics of the English task (i.e., syllable and phoneme deletion) and presented in order of increasing difficulty. The administration of the test was discontinued if the children made six consecutive errors.

#### Vocabulary

The Peabody Picture Vocabulary was used to measure English receptive vocabulary (PPVT-IV Form A; Dunn and Dunn, 2007) in grades 1 and 3. Each time a tester orally presented a target word, the child was required to point to one of four pictures that best corresponded to that word. Testing was discontinued when the child made eight or more errors in a set of 12.

The Échelle de Vocabulaire en Images Peabody (EVIP Form A; Dunn et al., 1993) was used to assess French receptive vocabulary in both grades. The examiner read a target word and the child was asked to identify the picture that best represented the word from a set of four pictures. Testing was discontinued after six errors were made on the previous eight consecutive items.

#### Word Reading Accuracy

Word reading accuracy in English was assessed in grades 1 and 3 with the Letter-Word Identification subtest from the Test of Achievement, Woodcock Johnson-III (WJ-III; Woodcock et al., 2001). Children were asked to read a series of 76 letters and words that were presented in order of increasing difficulty. Testing was discontinued after participants misread the six consecutive highest-numbered items on a given page.

French word reading accuracy was assessed using an experimental task (Au-Yeung et al., 2015). The test consists of 120 items arranged in 15 sets of eight words each. The children were asked to read the words accurately and fluently. Testing was discontinued when the children misread five or more words within a set of eight words. The total score represents the number of words read correctly.

#### Word Reading Fluency

Children’s word reading fluency in English was measured by the Sight Word Efficiency subtest of the Test of Word Reading Efficiency (TOWRE Form A; Torgesen et al., 1999) in grade 3. Children were provided with 45 s to quickly and accurately identify as many words as they could from a vertical list of 104 items. A parallel experimental measure was created to assess word reading fluency in French.

#### Reading Comprehension

The comprehension subtest (Level 3 Form S) of the Gates-MacGinitie Reading Tests (GMRT; MacGinitie et al., 2000) was used to assess children’s English reading comprehension in grade 3. Children were asked to read short passages and answer 48 corresponding multiple-choice questions. The score was the total number of correct answers. Level C Form 4 of the Gates-MacGinitie Reading Tests – Second Canadian Edition (MacGinitie and MacGinitie, 1992) was translated into French and administered in the same way as the English task.

## Results

To prepare the data for analyses, we first examined whether there was statistical support for merging the samples of children who spoke English as a primary language at home and those who were exposed to additional home languages into one sample. A Box’s *M* test using the grades 1 and 3 measures, indicated no significant difference in variance-covariance patterns between the two language groups on English, Box’s *M* = 40.88, *p* = 0.09, and French, Box’s *M* = 7.74, *p* = 0.99, reading and language measures. Based on these results, the two groups were combined to create one sample. Table 1 presents the mean raw scores, standard scores for standardized measures, standard deviations and reliability estimates for the entire sample on all English and French measures in grade 1 and grade 3.

**TABLE 1:** Means, standard deviations, and reliabilities for the total sample (*N* = 180) on English and French measures in grade 1 and grade 3.

**Measure**	***M***	***SD***	**Cronbach’s alpha**
**Grade 1**			
Age (in months)	80.36	4.18	
Non-verbal reasoning	4.49	3.59	0.86
English phonological awareness	15.45	6.59	0.94
English phonological awareness SS	11.34	3.06	
French phonological awareness	10.92	5.57	0.92
English vocabulary	122.86	20.23	0.95
English vocabulary SS	109.58	14.38	
French vocabulary	35.25	15.86	0.96
French vocabulary SS	69.02	14.17	
English word reading accuracy	32.43	11.10	0.95
English word reading accuracy SS	111.06	19.50	
French word reading accuracy	30.23	19.15	0.97
**Grade 3**			
Age (in months)	104.66	4.06	
English vocabulary	147.47	16.48	0.94
English vocabulary SS	108.47	13.30	
French vocabulary	66.57	26.20	0.97
French vocabulary SS	76.00	21.88	
English word reading accuracy	51.36	9.59	0.94
English word reading accuracy SS	109.47	14.61	
French word reading accuracy	65.40	25.49	0.98
English word reading fluency	60.74	15.31	0.97
English word reading fluency SS	95.83	17.20	
French word reading fluency	55.18	14.58	0.98
English reading comprehension	26.24	10.49	0.91
English reading comprehension SS	95.04	13.67	
French reading comprehension	17.79	7.39	0.84

*SS, standard score.*

We selected groups of comprehenders in grade 3 using separate regression techniques for English and French measures to predict children’s reading comprehension scores from age, non-verbal reasoning, word reading accuracy, and word reading fluency. These variables are correlated with reading comprehension (e.g., Deacon and Kirby, 2004; Lesaux et al., 2006) and have been widely used for identifying comprehender subgroups (Li and Kirby, 2014; Tong et al., 2014; D’Angelo and Chen, 2017). Together, the predictors explained a total of 43% of the variance in English reading comprehension and 37% of the variance in French reading comprehension. The observed reading comprehension scores were plotted against the standardized predicted scores. Children below the lower 65% confidence interval of the regression line were identified as poor comprehenders and those above the upper 65% confidence interval were identified as good comprehenders. Those children who scored within the 15% confidence interval were identified as average comprehenders. Children with very poor or good word reading skills (predicted value 1 SD above or below the mean) were not selected and excluded from analyses.

Through this regression method, we identified three groups of comprehenders in English (24 poor, 24 average, and 24 good) and three groups of comprehenders in French (24 poor, 24 average, and 24 good). Sixteen children out of the 24 poor comprehenders of English and 18 children out of the 24 poor comprehenders of French identified as English-speaking.^1^ The remaining children came from diverse linguistic backgrounds and were exposed to additional languages at home, including Russian, Hebrew, and Mandarin. A chi-square test of independence indicated a non-significant relationship between the children who spoke English as a primary language at home and those who were exposed to additional languages at home within the comprehender groups identified in English, χ^2^ (1, *N* = 72) = 3.11, *p* = 0.21, and in French, χ^2^ (1, *N* = 72) = 1.01, *p* = 0.61. Based on these results, and given that the children exposed to additional languages met the inclusion criteria (non-native speakers of French), they were retained in the sample.

We conducted multivariate analyses of variance (MANOVAs) to confirm the reading comprehension profiles of the English comprehender groups and to determine whether poor comprehenders differed from average and good comprehenders on English and French reading-related measures in grade 1 and grade 3. As illustrated in Table 2, there were no significant differences between the three groups on age, non-verbal reasoning, English and French word reading accuracy, and English and French elision in grade 1 and English and French word reading accuracy and fluency in grade 3 (all *p*s > 0.08). However, as expected, poor comprehenders differed significantly from average (*p* < 0.001) and good comprehenders (*p* < 0.001) on English and French reading comprehension in grade 3. Poor comprehenders also differed from average (*p* < 0.001) and good comprehenders (*p* < 0.001) on English vocabulary in grade 1 and grade 3. Similarly, French vocabulary distinguished poor comprehenders from average comprehenders in grade 1 (*p* < 0.05) and grade 3 (*p* < 0.01).

**TABLE 2:** Means (standard deviations) of poor, average, and good comprehenders selected with English measures on English and French reading and language variables in grade 1 and grade 3.

**Measure**	**Poor (*n* = 24)**	**Average (*n* = 24)**	**Good (*n* = 24)**		
	***M (SD)***	***M (SD)***	***M (SD)***	***F***	**Pairwise comparisons^a^**
**Grade 1**					
Age (in months)	80.06 (4.14)	80.78 (4.56)	81.42 (4.65)	0.49	
Non-verbal reasoning	3.37 (2.85)	5.08 (3.57)	5.29 (3.98)	1.81	
English phonological awareness	11.32 (3.22)	12.00 (11.92)	11.92 (3.13)	0.29	
French phonological awareness	10.96 (5.72)	11.08 (4.60)	12.33 (5.99)	0.46	
English vocabulary	107.25 (20.14)	128.50 (16.08)	137.58 (16.36)	18.73***	PC < AC < GC
French vocabulary	27.50 (11.22)	40.71 (17.04)	36.79 (17.22)	4.03*	PC < AC = GC
English word reading accuracy	30.95 (10.12)	33.54 (10.58)	33.46 (10.42)	0.40	
French word reading accuracy	33.22 (18.21)	31.37 (18.11)	32.50 (18.59)	0.61	
**Grade 3**					
Age (in months)	104.74 (4.41)	104.05 (4.32)	105.23 (3.63)	0.50	
English vocabulary	127.25 (20.28)	152.00 (10.67)	159.37 (9.40)	33.23***	PC < AC < GC
French vocabulary	50.05 (17.88)	73.79 (25.70)	67.96 (28.32)	5.34**	PC < AC = GC
English word reading accuracy	50.53 (10.75)	53.54 (7.45)	54.92 (5.86)	1.62	
French word reading accuracy	73.30 (28.40)	74.25 (25.98)	60.67 (19.26)	2.23	
English word reading fluency	59.21 (10.91)	61.96 (10.46)	66.79 (7.90)	2.10	
French word reading fluency	53.35 (15.67)	55.96 (11.22)	56.88 (8.24)	0.54	
English reading comprehension	15.84 (3.43)	27.25 (5.06)	40.67 (3.41)	198.51***	PC < AC < GC
French reading comprehension	15.09 (4.63)	15.08 (6.50)	23.42 (8.62)	11.89***	PC = AC < GC

^*a*^Equal sign indicates non-significant difference, and less-than symbol indicates *p* < 0.05 or less. *p < 0.05; **p < 0.01; ***p < 0.001.

For the comprehender groups identified using French measures, there were no significant differences between poor, average, and good comprehenders on age, non-verbal reasoning, and English and French phonological awareness in grade 1. Poor comprehenders differed significantly from average and good comprehenders on grade 1 measures of English (*p* < 0.01) and French vocabulary (*p* < 0.01) and English (*p* < 0.001) and French word reading accuracy (*p* < 0.001). In grade 3, English (*p* < 0.05) and French vocabulary (*p* < 0.001), English word reading accuracy (*p* < 0.001), English (*p* < 0.001) and French word reading fluency (*p* < 0.001), and English (*p* < 0.001) and French reading comprehension (*p* < 0.001) distinguished poor comprehenders from average and good comprehenders (Table 3).

**TABLE 3:** Means (standard deviations) of poor, average, and good comprehenders selected with French measures on English and French reading and language variables in grade 1 and grade 3.

**Measure**	**Poor (*n* = 24)**	**Average (*n* = 24)**	**Good (*n* = 24)**		
	***M (SD)***	***M (SD)***	***M (SD)***	***F***	**Pairwise comparisons^a^**
**Grade 1**					
Age (in months)	81.11 (4.83)	80.42 (3.79)	80.05 (3.99)	0.36	
Non-verbal reasoning	4.29 (3.21)	3.95 (3.12)	4.83 (3.99)	0.36	
English phonological awareness	13.33 (6.63)	15.41 (7.29)	17.78 (12.30)	2.11	
French phonological awareness	9.38 (4.47)	11.86 (6.44)	12.30 (5.41)	1.77	
English vocabulary	113.00 (20.24)	128.59 (14.92)	128.09 (14.96)	4.75**	PC < AC < GC
French vocabulary	28.14 (10.20)	39.77 (19.25)	44.22 (18.76)	5.35**	PC < AC = GC
English word reading accuracy	25.38 (9.12)	35.64 (8.23)	38.74 (11.69)	10.94***	PC < AC < GC
French word reading accuracy	23.81 (18.21)	30.91 (17.10)	33.45 (11.24)	7.29***	PC < AC = GC
**Grade 3**					
Age (in months)	105.07 (4.37)	104.65 (3.58)	105.61 (3.80)	0.35	
English vocabulary	141.67 (19.84)	150.29 (15.00)	154.05 (10.00)	3.65*	PC < AC < GC
French vocabulary	52.33 (19.96)	72.29 (25.07)	82.23 (23.86)	9.24***	PC < AC = GC
English word reading accuracy	47.95 (8.65)	54.37 (7.75)	57.32 (5.30)	9.08***	PC < AC < GC
French word reading accuracy	65.97 (27.63)	66.83 (20.81)	73.36 (20.99)	0.70	
English word reading fluency	56.57 (13.05)	68.21 (8.21)	68.50 (10.60)	8.75***	PC < AC < GC
French word reading fluency	50.76 (12.40)	60.92 (10.30)	62.00 (8.65)	7.75***	PC < AC < GC
English reading comprehension	20.52 (10.35)	30.21 (8.86)	38.77 (6.70)	23.45***	PC < AC < GC
French reading comprehension	11.10 (3.92)	18.37 (2.53)	28.82 (4.88)	114.77***	PC < AC < GC

^*a*^Equal sign indicates non-significant difference, and less-than symbol indicates *p* < 0.05 or less. *p < 0.05; **p < 0.01; ***p < 0.001.

Table 4 presents the prevalence rates of the overlap between comprehender groups in English and French. Of particular interest to this study was the number of children who were identified through the regression technique as poor comprehenders for both English and French relative to the entire sample. Three subgroups of reading comprehension difficulties in the two languages were considered: 10 children who were poor comprehenders in both English and French (PCB), 11 children who were poor comprehenders in English only (PCE), and 10 children who were poor comprehenders in French only (PCF). We selected an additional 10 children from among the good comprehenders in both English and French, matched on age and gender, to serve as the control group. In this way, we could compare the three groups of comprehenders to children who had average English and French word reading skills, but good comprehension in both English and French. There were no significant differences between the four groups on age (PCB: *M* = 104.26, *SD* = 3.97; PCE: *M* = 105.01, *SD* = 4.98; PCF: *M* = 104.01, *SD* = 4.40; Control: *M* = 105.02, *SD* = 3.46) and non-verbal reasoning (PCB: *M* = 3.80, *SD* = 3.01; PCE: *M* = 2.82, *SD* = 2.40; PCF: *M* = 3.80, *SD* = 2.25; Control: *M* = 5.00, *SD* = 4.14). Chi-square results demonstrated that the chance of poor comprehenders in English also being poor comprehenders in French was significantly above the baseline level, χ^2^ (1, *N* = 180) = 14.02, *p* < 0.001.

**TABLE 4:** The overlap and distribution of poor reading comprehension in English and French.

**Comprehender subgroup**	**Poor French**	**Average French**	**Good French**	**Not selected for analysis**	**Total**
	***n* (%)**	***n* (%)**	***n* (%)**	***n* (%)**	
Poor English	10 (41.7%)	2 (8.3%)	1 (4.2%)	11 (45.8%)	24
Average English	2 (8.3%)	0 (0%)	1 (4.2%)	21 (87.5%)	24
Good English	2 (8.3%)	3 (12.5%)	14 (58.3%)	5 (20.8%)	24
Not selected for analysis	10 (9.3%)	19 (17.6%)	8 (7.4%)	71 (65.7%)	108
Total	24	24	24	108	180

*χ^2^ (1, *N* = 180) = 14.02, *p* < 0.001.*

It should be noted that children identified as poor comprehenders in English only had not been selected for a comprehender status in French. Similarly, those identified as poor comprehenders in French only did not fit a comprehender group in English. Of the remaining children who were poor comprehenders identified in English, two were average comprehenders in French and one was a good comprehender in French. Of the remaining poor comprehenders identified in French, two were average comprehenders in English and two were good comprehenders English.

The next step in our analyses was to retrospectively examine the correlates of English and French reading comprehension difficulties for each of the three subgroups of poor comprehenders and the control group. We conducted separate MANOVAs, controlling for gender, for the English and French reading and language measures in each grade. Univariate analyses were computed for tasks tested at one time point only (i.e., English and French phonological awareness, English and French word reading fluency, and English and French reading comprehension). Table 5 shows the mean raw scores and standard deviations of the English and French reading and language measures for each group in grade 1 and grade 3, as well as comparisons across groups.

**TABLE 5:** Means (standard deviations) and comparisons of poor comprehenders in English and French, poor comprehenders in English only, poor comprehenders in French only, and controls on English and French measures in grade 1 and grade 3.

**Measure**	**PCB**	**PCE**	**PCF**	**Control group**		
	***M (SD)***	***M (SD)***	***M (SD)***		***F***	**Pairwise comparisons**
**Grade 1**						
English phonological awareness	10.00 (7.20)	14.00 (5.40)	13.70 (4.03)	18.20 (8.72)	2.62	
French phonological awareness	11.80 (6.70)	10.64 (5.45)	7.90 (3.60)	11.30 (6.04)	0.98	
English vocabulary	97.80 (19.80)	112.27 (19.04)	116.20 (19.64)	137.00 (13.57)	9.05***	PCB, PCE < C
French vocabulary	26.20 (11.19)	30.27 (11.44)	28.80 (11.24)	40.70 (20.40)	2.15	
English word reading accuracy	28.00 (8.40)	29.60 (10.44)	26.50 (11.41)	31.30 (11.01)	0.39	
French word reading accuracy	28.33 (19.47)	35.55 (17.77)	22.40 (19.29)	31.90 (13.25)	1.05	
**Grade 3**						
English vocabulary	120.00 (18.08)	127.73 (20.69)	145.80 (18.84)	158.50 (7.99)	13.47***	PCB < PCF, C; PCE < C
French vocabulary	47.20 (22.09)	69.91 (29.40)	48.20 (21.92)	73.10 (24.62)	3.04*	PCB < PCE, C; PCF < C
English word reading accuracy	47.22 (8.69)	51.90 (7.70)	46.40 (10.26)	55.50 (5.52)	2.64	
French word reading accuracy	73.30 (28.40)	74.25 (25.98)	60.67 (19.26)	65.90 (21.03)	0.89	
English word reading fluency	54.70 (20.82)	59.18 (12.98)	48.90 (11.21)	67.10 (11.08)	2.79	
French word reading fluency	50.40 (19.34)	54.36 (12.73)	45.20 (12.59)	58.80 (8.44)	0.17	
English reading comprehension	14.40 (2.59)	16.91 (2.21)	20.00 (11.17)	41.00 (3.53)	38.83***	PCB, PCE, PCF < C; PCB < PCF
French reading comprehension	11.90 (3.07)	15.82 (4.24)	10.50 (4.70)	28.00 (3.74)	37.84***	PCB, PCF < PCE, C; PCE < C

PCB, poor comprehenders in both English and French; PCE, poor comprehenders in English only; PCF, poor comprehenders in French only; C, control group. *p < 0.05; ***p < 0.001.

As expected, there were no significant differences between the four groups on the word reading measures used to select comprehender groups, word reading accuracy and fluency, for both English and French in grade 3, and consistent findings were revealed retrospectively for English and French word reading accuracy in grade 1. Similarly, the groups did not differ significantly on English and French phonological awareness in grades 1 and 3.

Results of univariate analyses showed that there was a significant overall group effect for English reading comprehension, *F*(3,41) = 38.83, *p* < 0.001, $\eta_{\text{p}}^{2}$ = 0.76 and French reading comprehension, *F*(3,41) = 37.84, *p* < 0.001, $\eta_{\text{p}}^{2}$ = 0.76. Tukey’s HSD *post hoc* comparisons showed that the PCB, PCE, and PCF groups performed worse than the control group on English reading comprehension in grade 3. The PCB group also scored significantly lower than the PCF group on English reading comprehension. For French reading comprehension in grade 3, all three poor comprehender groups (PCB, PCE, and PCF) scored significantly lower than the control group, with the PCF group also scoring lower than PCE group.

There was a significant overall group effect for English vocabulary, Wilks’ Λ = 0.41, *F*(6,70) = 6.64, *p* < 0.001, $\eta_{\text{p}}^{2}$ = 0.36, and French vocabulary, Wilks’ Λ = 0.29, *F*(6,72) = 3.60, *p* < 0.05, $\eta_{\text{p}}^{2}$ = 0.22. Univariate tests revealed that the four groups differed significantly in English vocabulary in grade 1, *F*(3,41) = 9.05, *p* < 0.001, $\eta_{\text{p}}^{2}$ = 0.43, and in grade 3, *F*(3,41) = 13.47, *p* < 0.001, $\eta_{\text{p}}^{2}$ = 0.54. Tukey’s HSD *post hoc* comparisons showed that children in the PCB and PCE groups scored significantly lower than the control group on English vocabulary in grades 1 and 3. However, in grade 3, the PCB group also scored lower than the PCF group on English vocabulary. The univariate tests for French vocabulary found no significant difference between groups on grade 1 French vocabulary, but there were significant group differences on French vocabulary in grade 3, *F*(3,41) = 3.04, *p* < 0.05, $\eta_{\text{p}}^{2}$ = 0.20. The *post hoc* test for French vocabulary showed that the PCB and PCF groups had significantly lower scores than control groups on French vocabulary in grade 3. The PCB group also had lower French vocabulary scores than the PCE group in grade 3.^2^

## Discussion

The aim of the present study was to investigate correlates and overlap of reading comprehension difficulties for bilingual poor comprehenders who are exposed to English, the societal language, and French, the language of classroom instruction. By identifying poor comprehenders of both English and French, we were able to determine to what extent poor comprehenders in English, a primary language, are also poor comprehenders in French, an additional language.

We found that there is a moderate degree of overlap in comprehension difficulties in English and French among poor comprehenders with equivalent amounts of exposure to French, with a prevalence rate of 41.7% in our sample. However, our findings also indicate that children who have reading comprehension difficulties in one language do not necessarily have difficulties in another. In addition, we found that English and French vocabulary was a strong and persistent indicator of reading comprehension difficulties in the same language for poor comprehenders of English, French, and both English and French.

Consistent with previous studies, results demonstrate that deficits in oral language are characteristic of children with poor reading comprehension (e.g., Nation et al., 2004, 2010; Catts et al., 2006). Building on previous work (D’Angelo et al., 2014), we found that poor comprehenders of English who received classroom instruction in French demonstrated concurrent vocabulary weaknesses in English and French relative to average and good comprehenders, despite comparable word decoding skills. Lower English vocabulary scores distinguished poor comprehenders from average and good comprehenders, whereas lower French vocabulary scores distinguished poor comprehenders from good comprehenders but not from average comprehenders. Similarly, for children identified in French, poor comprehenders differed from average and good comprehenders on English vocabulary, and from good comprehenders, but not average comprehenders on French vocabulary. These findings suggest that the average comprehenders in this study may have not yet reached a level of French proficiency needed to move beyond the performance of the poor comprehenders on French vocabulary. Vocabulary acquisition in French, an additional language, may be more challenging for immersion children because of their limited exposure to French outside of the classroom. Future research should include measures of cognitive abilities, such as phonological short-term memory that may be better at distinguishing group differences in the early grades (Farnia and Geva, 2011).

Regardless of English or French identification, the retrospective analyses indicated that differences between the three comprehender groups in English and French vocabulary were apparent in grades 1 and 3, with no group differences on English and French phonological awareness in grade 1. These findings clearly demonstrate that poor comprehenders’ oral language weaknesses are evident in the early stages of learning to read in both English and French. Although our study examines poor comprehenders in a bilingual context, these results are strikingly similar to findings reported by Catts et al. (2006) and Nation et al. (2010) and confirm that vocabulary weaknesses are apparent before poor comprehenders’ reading comprehension difficulties emerge. However, our study also found that there were differences between poor and average and good comprehenders identified in French on word reading measures in grade 1 and grade 3, indicating that different skills may lead to poor reading comprehension in English and French, and French reading comprehension may be more dependent on word level skills.

This study is the first to demonstrate that children with poor reading comprehension may experience difficulties with comprehension in English, in French, or in both English and French. Of these groups, children who were poor comprehenders in both English and French consistently scored the lowest on English vocabulary in grade 1 and grade 3 and in French vocabulary in grade 3 suggesting that severe English vocabulary weaknesses in poor comprehenders may contribute to comprehension difficulties in English and French. While there were no significant group differences found on phonological awareness, word reading and word fluency tasks, it is interesting to note that the poor comprehenders of both English and French, who were the poorest on English and French reading comprehension, also scored the lowest on all English and French reading and language measures in both grades 1 and 3. Results provide support for the linguistic interdependence hypothesis and suggest that children with poor reading comprehension in L1 may be at risk for being a poor comprehender in L2.

We found that 41.7% of children classified as poor comprehenders in grade 3 were poor comprehenders of both English and French. As expected, this overlap is less than reported in previous studies (e.g., Tong et al., 2017) in part due to differences in the approach to defining poor comprehender groups. More specifically, whereas most previous studies have defined poor comprehender groups based on a cut-off score on word reading, reading comprehension, or both, the present study utilized a regression method to identify poor comprehenders based on the relative discrepancy between wording reading, word reading fluency, and reading comprehension, while controlling for age and non-verbal reasoning, therefore, avoiding overidentification and narrowing the sample of children who qualify for poor comprehender status.

However, it could be argued that the overlap between English and French poor comprehender status should be greater given that English and French are alphabetic orthographies and share many linguistic features. It is worth noting that children in this study had been receiving classroom instruction in French for approximately 3 years at the time of comprehender classification. It is possible that children’s poor comprehension in French would have been more apparent had they been exposed to French for a longer period of time. This explanation is consistent with that of previous research, which has demonstrated that relative to poor decoders, poor comprehenders’ difficulties with reading comprehension emerge around the age 10, when performance in reading comprehension is equally accounted for by oral language and decoding skills (e.g., Elwér et al., 2013). Therefore, it seems plausible that there would be a greater overlap of poor comprehender status with more exposure to the French language in spoken and written form. Further research is needed to investigate the overlap of English and French reading comprehension difficulties in the later elementary grades, as decoding becomes more automatized and greater variance is accounted for by oral language skills.

The current study examined the learning needs of poor comprehenders in immersion education and has important implications for the assessment and remediation of reading comprehension difficulties in emerging bilingual learners. Our findings demonstrate that poor comprehenders exhibit pervasive oral language difficulties from the onset of reading that manifest similarly in English, their primary language, and French, the language of instruction. Furthermore, the results suggest that it is possible for children to experience poor reading comprehension in one language but be relatively good at comprehension in another language. Since many children begin French immersion with limited levels of French language proficiency, it is beneficial to gather information on children’s reading and language abilities with parallel measures in English and French. Limiting assessment to French, an additional language, may underestimate children’s reading and language ability or misattribute reading difficulties to a lack of French proficiency (Geva and Herbert, 2012).

This research also suggests that intervention strategies should be targeted at poor comprehenders’ underlying language difficulties regardless of language of instruction. While there have been relatively few intervention studies with poor comprehenders, existing studies have shown that intervention practices that promote oral language skills and text comprehension strategies are effective supports for monolingual children with poor reading comprehension (Snowling and Hulme, 2012). Evidently, there is a need for future intervention research that fosters the development of children’s oral language skills in immersion programs.

There are some limitations of the current study that should be noted. First, the sample of poor comprehenders identified within the three subgroups (i.e., PCB, PCE, PCF) was small, which limits the generalizability of our findings. However, obtaining a large sample of poor comprehenders is particularly challenging in a bilingual educational context. Our study is among the few longitudinal studies that have examined bilingual poor comprehenders’ reading and language skills in both languages over time. Given the attrition of students in French immersion (e.g., Chen et al., 2019) and the prevalence rate of poor comprehenders in middle elementary years at approximately 10% (e.g., Nation and Snowling, 1998; Clarke et al., 2010), our sample size may be considered representative of poor comprehenders in a bilingual context. Nevertheless, larger sample sizes for the subgroups of poor comprehenders would benefit future work.

Reading comprehension is a complex process that involves the coordination of various skills that are assessed differently across measures of reading comprehension. In the present study, we used a single standardized measure of reading comprehension. Although the use of this standardized test makes our sample of poor comprehenders comparable to those in the existing monolingual literature (e.g., Tong et al., 2014), results reported in this study need to be replicated with more varied reading comprehension measures to disentangle whether poor comprehenders score low on reading comprehension because they do not understand the text or because they are unable to read the question. Similarly, the use of a single measure of vocabulary knowledge may not fully capture the influence of other language skills on reading comprehension, such as vocabulary depth, listening comprehension, morphological awareness, and inference (Nation and Cocksey, 2009; D’Angelo and Chen, 2017).

Another limitation is that approximately 25% of the children identified as poor comprehenders in either English, French, or both were exposed to another language at home in addition to English. While this sample is representative of students enrolled in French immersion programs in Canada, there is a need for further research to explore whether significant differences exist between children identified as poor comprehenders from English monolingual backgrounds and those who speak additional languages.

Finally, there is some difficulty in interpreting poor comprehender status in French only, particularly for children in this study who grew up in an English-speaking community. Poor reading comprehension in French may not be attributed to a language impairment or limited proficiency in French but associated with children’s lack of motivation to learn in an L2. Evidently, there is a need for further research to explore the role of motivation in L1 and L2 reading comprehension for children enrolled in immersion programs.

Taken together, the present study demonstrates that poor comprehenders experience similar and persistent difficulties with components of language in both English, a primary language, and French, an additional language, that are present in the early stages of reading development, and therefore, likely indicators of later reading comprehension difficulties in both languages. These results also show while there is a moderate degree of overlap in English and French reading comprehension difficulties, not all poor comprehenders of English are poor comprehenders of French, suggesting that somewhat different skills may be involved in comprehending text in English and French.

## Data Availability Statement

The datasets generated for this study are available on request to the corresponding author.

## Ethics Statement

The studies involving human participants were reviewed and approved by University of Toronto Research Ethics Board. Written informed consent to participate in this study was provided by the participants’ legal guardian/next of kin.

## Author Contributions

ND’A and XC contributed to the conception and design of the study. ND’A and KK organized data collection and managed the database and performed the statistical analyses. ND’A wrote the first draft of the manuscript. KK and XC wrote sections of the manuscript. All authors contributed to manuscript revisions and read and approved the submitted version.

## Conflict of Interest

The authors declare that the research was conducted in the absence of any commercial or financial relationships that could be construed as a potential conflict of interest.

## Footnotes

1 For children to be classified as English-speaking, parents had to indicate that English was spoken in the home environment 50% of the time or more.

2 Due to the small group sizes, equivalent non-parametric tests were calculated for each analysis. The Kruskal–Wallis test, used for comparing two or more independent samples, confirmed our parametric results.
